# Supplementary material for: A high-resolution mRNA expression time course of embryonic development in zebrafish
Source: eLife. 2017 Nov 16;6:e30860. doi: 10.7554/eLife.30860 (PMC5690287; doi:10.7554/eLife.30860)
Supplement: Supplementary file 6. [file elife-30860-supp6.zip › biolayout-clusters-files/Cluster170-genes.html]

Cluster170


# Cluster170: Genes

| Ensembl ID | Gene Name | Chr | Start | End | Biotype |
| --- | --- | --- | --- | --- | --- |
| ENSDARG00000068759 | ENSDARG00000068759 | 5 | 13977065 | 14000405 | protein\_coding |
| ENSDARG00000075033 | GTPBP8 | 9 | 27518894 | 27526439 | protein\_coding |
| ENSDARG00000105195 | dnajc21 | 21 | 11087186 | 11106770 | protein\_coding |
| ENSDARG00000017359 | mettl8 | 9 | 3547662 | 3563495 | protein\_coding |
| ENSDARG00000009214 | mfsd13a | 12 | 33793492 | 33814140 | protein\_coding |
| ENSDARG00000101024 | nufip1 | 6 | 59739185 | 59746399 | protein\_coding |
| ENSDARG00000051902 | prmt7 | 25 | 14312536 | 14328006 | protein\_coding |
| ENSDARG00000052375 | zgc:66455 | 1 | 52817984 | 52833422 | protein\_coding |
